# Taxonomía de los hongos: un rompecabezas al que le faltan muchas piezas

**DOI:** 10.7705/biomedica.7052

**Published:** 2023-08-31

**Authors:** Álvaro León Rúa-Giraldo

**Affiliations:** 1 Escuela de Microbiología, Universidad de Antioquia, Medellín, Colombia Universidad de Antioquia Universidad de Antioquia Medellín Colombia

**Keywords:** hongos, clasificación, filogenia, evolución biológica, código de barras del ADN taxonómico, Fungi, classification, phylogeny, biological evolution, DNA barcoding, taxonomic

## Abstract

Los hongos son organismos polifacéticos presentes en casi todos los ecosistemas de la tierra, donde establecen diversos tipos de simbiosis con otros seres vivos. A pesar de ser reconocidos por los humanos desde la antigüedad -y de la cantidad de trabajos que han profundizado sobre su biología y ecología-, aún falta mucho por conocer sobre estos organismos. Algunos de los criterios que clásicamente se han utilizado para su estudio, hoy resultan limitados y hasta cierto punto permiten un agrupamiento de los aislamientos según algunas características, pero generan confusión en su clasificación y, más aún, cuando se pretende comprender sus relaciones genealógicas.

Los caracteres fenotípicos no son suficientes para identificar una especie de hongos y, menos aún, para construir una filogenia amplia o de un grupo particular. Hay grandes vacíos que hacen que los árboles generados sean inestables y fácilmente debatidos. Para los profesionales de la salud, parece que la identificación de los hongos hasta niveles inferiores como género y especie es suficiente para elegir el tratamiento más adecuado para su control, comprender la epidemiología de los cuadros clínicos asociados y reconocer los brotes y los factores determinantes de la resistencia a los antimicrobianos. No obstante, la ubicación taxonómica dentro del reino permitiría establecer relaciones filogenéticas entre los taxones fúngicos, facilitando la comprensión de su biología, su distribución en la naturaleza y la evolución de su potencial patogénico. Los avances de las técnicas de biología molecular y las ciencias de la computación en los últimos 30 años han permitido cambios importantes dirigidos a establecer los criterios para definir una especie fúngica y alcanzar una construcción filogenética más o menos estable. Sin embargo, el camino por recorrer aún es largo, y supone un trabajo mancomunado de la comunidad científica a nivel global y el apoyo a la investigación básica.

## Abstract (en)

Fungi are multifaceted organisms found in almost all ecosystems on Earth, where they establish various types of symbiosis with other living beings. Despite being recognized by humans since ancient times, and the high number of works delving into their biology and ecology, much is still unknown about these organisms.

Some criteria classically used for their study are nowadays limited, generating confusion in categorizing them, and even more, when trying to understand their genealogical relationships. To identify species within Fungi, phenotypic characters to date are not sufficient, and to construct a broad phylogeny or a phylogeny of a particular group, there are still gaps affecting the generated trees, making them unstable and easily debated. For health professionals, fungal identification at lower levels such as *genus* and species, is enough to select the most appropriate therapy for their control, understand the epidemiology of clinical pictures associated, and recognize outbreaks and antimicrobial resistance. However, the taxonomic location within the kingdom, information with apparently little relevance, can allow phylogenetic relationships to be established between fungal taxa, facilitating the understanding of their biology, distribution in nature, and pathogenic potential evolution. Advances in molecular biology and computer science techniques from the last 30 years have led to crucial changes aiming to establish the criteria to define a fungal species, allowing us to reach a kind of stable phylogenetic construction. However, there is still a long way to go, and it requires the joint work of the scientific community at a global level and support for basic research.

Cada vez los hongos reciben más atención y pasan a ocupar el sitio de honor que les corresponde por ser los moldeadores de la vida en la tierra. Es indiscutible su importancia ecológica y para la obtención de productos útiles para el humano, pero no se pueden menospreciar sus efectos dañinos en los seres vivos de todos los reinos. Las pérdidas económicas por afectación de cultivos de alimentos, el deterioro que generan sobre prácticamente cualquier material, la contaminación de ambientes extramurales e intramurales y los procesos de enfermedad en humanos y otros animales, hacen que sean nombrados a diario en los medios de comunicación masivos. Por ejemplo, *Candida auris,* como agente patógeno de fácil diseminación en ambientes hospitalarios y con gran resistencia a los antimicóticos de uso rutinario; los mucorales y su oportunismo en los pacientes con COVID-19, y la adquisición de resistencia cruzada a los antifúngicos por su uso indiscriminado en áreas de cultivo de algunos mohos ambientales que pueden infectar a los humanos, han activado las alarmas de los sistemas de salud mundial.

No obstante, a pesar de que los hongos son reconocidos por los humanos desde la prehistoria, es relativamente poco lo que se sabe de ellos. En esta revisión se pretende abordar los conceptos más recientes sobre la definición de una especie dentro de los hongos, y los métodos que se han utilizado para identificarlos y organizarlos de acuerdo con su ascendencia evolutiva. Al final, se presenta la clasificación que, por su solidez y poca complejidad, describe de una manera más sencilla el árbol de la vida del reino Fungi *(Fungal Tree of Life,* FToL).

El reino de los hongos es el menos conocido de los tres "grandes" reinos eucarióticos después de los animales y las plantas, a pesar de ser el segundo grupo de organismos más rico en especies después de los animales ^1^. En la naturaleza, los hongos son los principales degradadores de la materia orgánica, responsables de convertir los organismos muertos en pequeños bloques de nutrientes que otros seres vivos pueden utilizar. Es así como en las selvas tropicales, los hongos degradan, aproximadamente, el 50 % de la materia vegetal y animal muerta para evitar su acumulación y que asfixie a los demás seres vivos ^2^.

Sin embargo, los hongos también generan daño. Su capacidad de adaptación hace que puedan colonizar prácticamente todo ambiente sobre la tierra, contaminando y destruyendo todo a su paso ^3^. Algunos hongos son importantes fitopatógenos capaces de causar grandes pérdidas económicas por contaminación de los cultivos de importancia agrícola ^4^; también, pueden generar problemas de salud en humanos y animales, como procesos infecciosos, alérgicos e intoxicaciones ^5^. Los hongos patógenos y los oportunistas causan más de mil millones de infecciones humanas al año, con más de 1,6 millones de muertes ^6^.

Es dentro de este grupo donde recientemente se ha manifestado gran confusión debido a los constantes cambios taxonómicos de agentes patógenos reconocidos; algunos han pasado de ser una especie a un complejo de especies crípticas, con diferencias en su grado de virulencia y sensibilidad a los antifúngicos e, incluso, con cambio de género o una nueva denominación específica.

La identificación correcta de los hongos reviste gran importancia práctica, no sólo en el ámbito clínico humano, sino también, en patología vegetal, biodeterioro, biotecnología y estudios medioambientales. En la actualidad, esto cobra más relevancia cuando se evidencia el efecto del cambio climático sobre el comportamiento ecológico de los hongos, la epidemiología de las micosis ^7^^)^ y de los agentes patógenos de los cultivos ^8^, y la aparición más frecuente de hongos resistentes a los antifúngicos de uso común ^9^. Además, está el creciente interés por conocer la biodiversidad en el planeta para aprovechar su arsenal biotecnológico ^10^ o por describir nuevas especies antes que la evolución las elimine para siempre ^11^.

La taxonomía pretende colaborar en la clasificación y organización de los hongos, pero esta disciplina, dinámica y progresiva que se apoya en los avances científicos y tecnológicos, se ve obligada a la constante revisión y actualización, y origina -a veces de manera más acelerada de lo que es posible asimilar- cambios nomenclaturales en la construcción del árbol de la vida del reino Fungi ^12^^,^^13^.

## ¿Qué se considera una especie fúngica?

Una especie es definida como la unidad básica de clasificación biológica, pero no es un dato estático, sino que debe probarse cuando se disponga de nueva información. En la literatura biológica hay más de 30 conceptos de especies ^14^, incluidos los de uso común, como los morfológicos, los ecológicos, los fenotípicos, los biológicos, los de reconocimiento, los evolutivos, los de conglomerado genotípico y los de especies filogenéticas ^15^^,^^16^.

Entre los micólogos, se han utilizado diferentes conceptos para definir las especies fúngicas ^15^^-^^17^. El enfoque más clásico, con muchas disparidades taxonómicas, es el concepto morfológico (fenotípico) que define una especie con base en sus características morfológicas e, idealmente, por las diferencias entre ellas. Otros conceptos de amplio uso, principalmente en estudios ambientales y en fitopatología, son el politético, el ecológico y el biológico.

Los avances en biología molecular y su aplicación para el estudio de los genomas, además de las herramientas bioinformáticas que permiten análisis filogenéticos de mayor validez estadística, generaron el concepto de especie filogenética, ampliamente acogido por la comunidad científica, pero sin lograr su consenso ^17^. Sin embargo, este concepto ha tenido gran impacto en el posicionamiento taxonómico, pero con un efecto sutil en la clasificación de aquellos microorganismos asociados con enfermedades. No obstante, aunque los estudios basados en la filogenia genómica ofrecen nuevas perspectivas sobre la clasificación a niveles taxonómicos superiores, el reconocimiento de las especies y sus relaciones siguen siendo muy controvertidos y están sujetos a diferentes interpretaciones.

Esta diversidad de conceptos genera cierto caos en el momento de definir una especie, debido a que todos se siguen empleando y no hay consenso sobre cuál o cuáles deben ser abandonados o cuál es el concepto que debería ser seguido por todo taxónomo de hongos ^18^. Además, los hongos son un grupo demasiado diverso, lo que conlleva una alta plasticidad fenotípica originada por la naturaleza polifilética de las especies, los efectos del ambiente sobre su genoma o ambos. Por el contrario, los caracteres morfológicos, fisiológicos, ecológicos y hasta los de homología de secuencias, pueden compartirse entre especies no tan relacionadas filogenéticamente ^19^.

Además, el mencionado creciente interés por conocer la biodiversidad, aunado al rápido desarrollo de las ciencias ómicas, ha incrementado de manera exponencial el número de posibles especies "nuevas" en las bases de datos de hongos, especialmente por estudios en lugares poco explorados ^19^, muchas de las cuales son sinónimos para especies ya reconocidas fenotípicamente, pero que no contaban con información de su genoma. Asimismo, un número muy importante de datos de secuencias parciales o totales de genomas han sido depositados sin seguir ninguna directriz consensuada, lo cual genera ruido y confusión ^20^^,^^21^.

Frente a este panorama tan agobiante, se han presentado varias propuestas que buscan establecer tales directrices con el fin de "normalizar" la manera de completar la información de los taxones ya reconocidos o la inclusión de otros nuevos en las bases de datos. En este sentido, Vellinga *et al.*^18^ propusieron unos criterios para introducir nuevos géneros fúngicos. No obstante, para el nivel de especie, no ha sido posible alcanzar un acuerdo entre las numerosas revisiones y propuestas ^15^^,^^17^.

## ¿Cómo identificar una especie de hongo?

Las personas dedicadas al estudio y, específicamente, a la identificación de hongos, tradicionalmente han empleado los caracteres observables como criterio principal ^22^; sin embargo, este enfoque fenotípico ha sido muy criticado por su falta de terminología estandarizada y estable, y por su gran subjetividad. Además, se ha considerado que algunas características fenotípicas son inestables y dependen de las condiciones ambientales, como ocurre con el crecimiento en un cultivo artificial. Una clara limitación de los enfoques fenotípicos es que no pueden aplicarse a hongos que no crecen en cultivo ^23^. Además, los caracteres morfológicos son consecuencia de la evolución, por lo que su uso dentro de la sistemática fúngica como criterio de identificación, puede llevar a errores debido a procesos genéticos como la hibridación, la especiación críptica y la evolución convergente ^24^.

No obstante, las características macromorfológicas y micromorfológicas se han empleado -en algunos casos con mucho éxito- para la identificación y la clasificación de la mayoría de los hongos consignados en las bases de datos; incluso, las claves dicotómicas para el reconocimiento de especies basadas en características morfológicas se continúan utilizando en el diagnóstico clínico humano y en fitopatología ^25^. Tales características pueden ser de gran utilidad para la clasificación a nivel de orden y familia, pero suelen ser insuficientes para determinar niveles inferiores como género y especie ^26^. Otras características han sido introducidas progresivamente para ayudar a definir las especies de hongos, entre las que se incluyen factores ecológicos (abióticos y bióticos), fisiológicos, bioquímicos, reproductivos y genéticos ^27^.

La aplicación de las técnicas de biología molecular para el estudio del genoma de los hongos ha tenido gran impacto en la delimitación de especies y en la reevaluación de los caracteres diagnósticos (morfológicos, ecológicos y biológicos); además, ha hecho que la práctica de identificar y clasificar solo por la morfología ya no sea aceptable, según el último código internacional de nomenclatura para algas, hongos y plantas (CINB) ^28^.

Entre los métodos basados en secuencias de ADN para identificar especies de hongos, se resalta el código de barras de ADN, tecnología en la que se compara una o varias secuencias desconocidas (por lo general, una región corta de ADN, de 400 a 800 pares de bases) con una base de datos de secuencias reconocidas y depuradas de ADN ^29^. El uso de las secuencias de ADN fúngico amplificadas con iniciadores para las dos subunidades (subunidad grande o LSU-26S o 28S y subunidad pequeña o SSU-18S) del ARN ribosómico, además de toda la región del espaciador transcrito interno (ITS1, 5.8S, ITS2), inició una nueva era en la identificación de secuencias filogenéticas moleculares en el reino Fungi ^30^.

En el 2011, durante el simposio *Amsterdam Declaration on Fungal Nomenclature*^31^, se seleccionó la región conocida de manera general como "región ITS", como el marcador oficial del código de barras para los hongos ^32^. Varias técnicas novedosas de secuenciación, como la secuenciación de próxima generación *(next-generation sequencing,* NGS), ONT MinlON nanopore y Pac Bio *sequencing,* permitieron que el código de barras del ADN sea más seguro, rápido, confiable y económico para la identificación de los hongos ^33^^,^^34^, e incrementaron vertiginosamente las secuencias de ITS disponibles en las bases de datos. Específicamente, en la versión 9.0 del 2022 de la base UNITE (https://unite.ut.ee/) se contabilizaban 6'441.764 secuencias de referencia ITS de hongos pertenecientes a 290.922 hipótesis de especies.

Estas especies hipotéticas se refieren a aquellas que no cuentan con la revisión taxonómica que debería acompañar a una especie e incluyen a los taxones conocidos como *dark taxa* o taxones oscuros, que podrían ser ya conocidos, pero que no se contaba con su secuencia ITS en la base de datos, o que podrían corresponder a taxones nuevos no caracterizados ^35^. Debido a la variación intragenómica de las secuencias ITS de muchos hongos (alrededor del 70 % de los incluidos en las bases de datos) ^35^^,^^36^, dichas secuencias son útiles en la identificación de un gran número de especies. Sin embargo, se ha demostrado que tal variabilidad no es tan prevalente o puede estar ausente en algunos géneros (~3-5 % de 127 hongos pertenecientes a Ascomycota y Basidiomycota), principalmente en aquellos con muchas especies, como *Aspergillus, Alternaría, Cladosporium, Penicillium* y *Trichoderma*^37^.

Otro aspecto limitante sobre el uso de las secuencias ITS (o de cualquier secuencia de referencia) para la identificación de las especies de hongos, es la asignación de valores de corte, es decir, qué tanto porcentaje de diferencia en la composición del ADN puede considerarse suficiente para separar dos especies ^38^. No es posible aplicar un solo valor para todas las especies, incluso dentro de un mismo género, y más aún en aquellos con gran frecuencia de especies crípticas ^39^. También, se reconoce la dificultad para obtener secuencias ITS de alta resolución directamente por PCR y secuenciación Sanger de productos de PCR, debido a la heterogeneidad entre las copias del ITS dentro del grupo de genes ribosómicos de una misma cepa ^40^. Además, el análisis de especies tipo depositadas en "fungarios", o colecciones de hongos, ha demostrado la incapacidad de los iniciadores universales ITS para amplificar alrededor del 10 % de los hongos estudiados ^32^^,^^41^.

Para estos géneros de difícil delimitación a nivel de especie con este marcador o cuando se requiera solo llegar hasta niveles taxonómicos intermedios como familia o género, puede recurrirse a la amplificación y secuenciación de la región LSU (subunidad ribosómica grande del ARN) con sus dominios hipervariables D1 y D2 para establecer relaciones entre especies, o combinada con la región ITS para llegar a nivel de especie ^42^. Este es el juego de secuencias más utilizado en estudios de sistemática de hongos, como el ensamblaje del árbol de la vida del reino Fungi ^43^.

La incapacidad del marcador ITS para diferenciar las especies de algunos géneros, incluyendo importantes agentes patógenos oportunistas de animales y plantas, ha obligado a los micólogos a buscar alternativas. Los otros genes elegidos codifican para proteínas y poseen regiones intrónicas (no codificantes) que suelen evolucionar a un ritmo más rápido en comparación con los ITS ^44^; suelen presentarse como copia única en el genoma, lo que permite un fácil reconocimiento de su homología y convergencia. También, poseen una longitud menos variable debido a que acumulan menos mutaciones en sus exones y, comparados con los genes ribosómicos, son más fáciles de alinear porque contienen menos ambigüedad debido a las restricciones de codones ^44^^,^^45^. Hay un consenso emergente sobre la utilidad del factor de elongación de la traducción 1-α (Tef7) como un código de barras excelente para la mayoría de los hongos estudiados ^46^.

Otros marcadores con aportes mayores en la identificación de especies de ciertos linajes incluyen la subunidad más grande *(RPB1)* y la segunda más grande de la ARN polimerasa ^45^^,^^46^, la β-tubulina (Tub2/BenA), la sexta subunidad de la ATP sintasa (ATP6) y el gen parcial de la calmodulina *(CaM)* de importancia para el estudio de Eurotiales, orden que incluye a *Aspergillus* y *Penicillium*^46^. También, la proteína de mantenimiento del minicromosoma (MCM7) es promisoria para inferir relaciones filogenéticas de nivel superior e inferior ^47^.

Otros códigos de barras también específicos de taxón, son TOP1 (topoisomerasa 1) y PGK (fosfoglicerato cinasa) útil en la identificación de especies de Ascomycota, principalmente *Fusarium* y *Penicillium;* LNS2 (proteína hipotética) para miembros de Pucciniomycotina, ACT (Ɣ-actina) para Sordariomycetes, Dothideomycetes y otros fitopatógenos; y COX1 (subunidad de la citocromo oxidasa I) utilizada en la identificación de los hongos del suelo. Estos marcadores podrían servir para complementar la información cuando los ITS y Tef1 sean insuficientes ^45^^,^^46^^,^^48^.

## ¿Cuántas especies de hongos existen?

El número de hongos siempre ha sido un tema intrigante, por lo que muchos grupos, principalmente de micólogos taxónomos o que trabajan con biodiversidad, han propuesto modelos para estimar su número real.

Las primeras estimaciones se basaron en el número de hongos registrados en plantas concretas. Bisby y Ainsworth, en 1943 ^49^, estimaron el número de hongos en unos 100.000; no obstante, quizá el punto de partida fue el trabajo de Hawksworth en 1991 ^50^, en el que estimaba que había 1,5 millones de especies, aproximadamente, basado -entre otros parámetros-en la existencia de unos seis taxones únicos para cada especie veget al.

Los avances recientes en las tecnologías de secuenciación del ADN han dado lugar a cambios en las estimaciones del número de especies fúngicas. Blackwell, en el 2011, con los hallazgos de los estudios de secuenciación de próxima generación, asumió que el número de especies fúngicas oscilaba entre 3,5 y 5,1 millones ^51^. No obstante, estas estimaciones se basan en el estudio de ciertas plantas huéspedes y se reconoce que muchos hongos no tienen una especificidad de huésped aparente y son bastante ubicuos, lo que seguro lleva a subestimar el número real de especies.

En 2017, Hawksworth y Lücking consideraron de 2,2 a 3,8 millones como las cifras más acertadas, también partiendo de extrapolaciones de las proporciones planta:hongo, y teniendo en cuenta las especies crípticas, las tasas y los patrones con los que se están describiendo nuevas especies, los nichos inexplorados y las especies basadas en el ADN recuperado de muestras ambientales ^52^. Con los datos generados recientemente a partir de estudios independientes y dependientes de cultivos, se calculó que las especies de hongos en la tierra ascienden a 12 (11,7-13,2) millones ^19^.

Recientemente, Baldrian *et al.*^53^ analizaron las secuencias de más de 200 estudios publicados hasta el 2019, en los que se emplearon secuenciación de ADN de alto rendimiento para obtener más de 250 millones de secuencias ITS2, y estimaron que la riqueza total de taxones fúngicos no sintónicos es de 6,28 millones. No obstante, para el 2021, en las bases de datos reconocidas [Index Fungorum (https://www.indexfungorum.org/) y Mycobank (https://www.mycobank.org/)], solo hay un poco más de 150.000 especies aceptadas, aunque estas cifras seguirán cambiando a medida que se diseñen nuevas metodologías de fácil ejecución, idealmente económicas, y cuando se realicen estudios en nuevos ambientes.

Por el momento, es necesario pensar en dónde pueden encontrarse las especies de hongos que aún faltan para completar el árbol de la vida del reino Fungi ^39^. Hawksworth y Rossman ^54^ consideraron que los hongos no conocidos residen en hábitats ya conocidos y explorados, pero, simplemente, no es posible su recuperación y estudio con los métodos hasta ahora empleados. También, deben encontrarse en nichos no estudiados, debido a que, como se ha demostrado en algunos estudios, los microorganismos presentan patrones biogeográficos ^55^.

Muchas de las piezas que le faltan a la construcción del árbol de la vida fúngica no son fácilmente recuperables con las técnicas clásicas basadas en cultivo, pero su identificación solo a partir de secuencias denominas unidades taxonómicas moleculares operativas sin nombre *[Molecular Operational Taxonomic Unit* (MUTO)] aún no es aceptada por los estatutos que rigen el actual CINB (56); se consideran taxones oscuros, reconocidos en todos los linajes fúngicos principales ^57^ y, en algunos casos, los únicos miembros descritos para linajes importantes, principalmente de los grupos basales ^58^.

## Taxonomía fúngica: darle orden al caos

En la actualidad, el interés no solo yace en la identificación y reconocimiento de las especies, sino, también, en el establecimiento de relaciones entre ellas y en organizarlas en grupos definidos para construir el árbol de la vida de los diferentes reinos de los seres vivos ^59^. La taxonomía fúngica busca descubrir, describir y clasificar todas las especies de hongos, además de proporcionar herramientas para su identificación ^20^.

La biología molecular, desde su llegada, ha reorganizado constantemente el árbol de la vida fúngico, gracias al estudio de secuencias de ADN e, inclusive, del genoma completo, que permiten inferir relaciones filogenéticas entre linajes de hongos y facilitan la detección de especies crípticas con caracteres morfológicos o fisiológicos similares ^27^^,^^37^.

En la actualidad, hay muchos géneros y especies para los cuales no se ha podido resolver su posición taxonómica y para los que nuevos datos moleculares (incluyendo nuevas secuencias informativas o análisis de genoma completo) deben ser estudiados con un enfoque polifásico ^44^. Existen varias bases de datos con secuencias de ADN para la identificación de especies de hongos y organismos relacionados, y la generación de árboles filogenéticos y filogenómicos. Las más empleadas se presentan en las revisiones de Jayasiri *et al.*^60^, Prakash *et al.*^61^ y Raja *et al.*^37^.

En general, quienes trabajan en la taxonomía de hongos sienten la necesidad de contar con una base de datos unificada que relacione toda la información actual o que tenga una interacción directa entre las diferentes bases de datos para la actualización de los nuevos taxones y completar vacíos importantes en en su conocimiento.

## El árbol de la vida de los hongos

A pesar de que los micólogos adoptaron desde muy temprano la sistemática molecular para el descubrimiento y la clasificación de los hongos, la organización en su árbol de la vida sigue siendo muy cambiante, principalmente, a nivel de las ramas más basales. Esto se debe a la naturaleza oculta y microscópica de muchos taxones que no han sido considerados o cuentan con muy pocos representantes para la generación de ramas sólidas dentro de las construcciones filogenéticas.

En las últimas décadas se han producido cambios drásticos en la taxonomía superior de los hongos, evidenciados en la triplicación de los filos fúngicos, que han pasado de cuatro a doce o hasta 19 ^62^. Dos ingentes esfuerzos separados cuyo objetivo es aumentar drásticamente el muestreo de genomas para la construcción del árbol de la vida fúngico son el Proyecto *"1000 Fungal Genomes"*(http://1000.fungalgenomes.org/home), en el cual se propuso la secuenciación de 1.000 genomas fúngicos de todos los niveles del árbol, pero con mayor muestreo de linajes poco representados o ausentes en las bases de datos genómicas ^63^, y el conocido como *"Y1000+ Project* (https://y1000plus.wei.wisc.edu), que propuso secuenciar los genomas de las 1.000 especies, aproximadamente, conocidas de levaduras (filo Ascomycota, subfilo Saccharomycotina).

Estos dos proyectos, más un número significativo de trabajos de grupos de todo el planeta, dejan a disposición de los taxónomos 2.420 secuencias de genomas completos de hongos a mediados de mayo de 2023 (https://mycocosm.jgi.doe.gov/mycocosm/home) (cuadro 1).

**Cuadro 1:** Número de especies de hongos con genomas secuenciados, organizados por linajes superiores (https://mycocosm.jgi.doe.gov/mycocosm, revisado a mayo 23 de 2023)

Grupo taxonómicos	Especies con genomas completos secuenciados
*Pucciniomycotina*	64
*Ustilaginomycotina*	39
*Agaricomycetes*	491
*Dacrymycetes*	9
*Tremellomycetes*	33
*Wallemiomycetes*	2
*Pezizomycetes*	74
*Orbiliomycetes*	3
*Eurotiomycetes*	414
*Dothideomycetes*	219
*Lecanoromycetes*	12
*Leotiomycetes*	84
*Sordariomycetes*	530
*Xylonomycetes*	2
*Saccharomycotina*	156
*Taphrinomycotina*	14
*Glomeromycotina*	13
*Mortierellomycotina*	85
*Mucoromycotina*	77
*Entomophthoromycotina*	5
*Kickxellomycotina*	13
*Blastocladiomycota*	4
*Chytridiomycetes*	24
*Monoblepheridomycetes*	2
*Neocallimastigomycetes*	12
*Microsporidia*	23
*Cryptomycota*	3
Total	2.420

**Figura 1:**
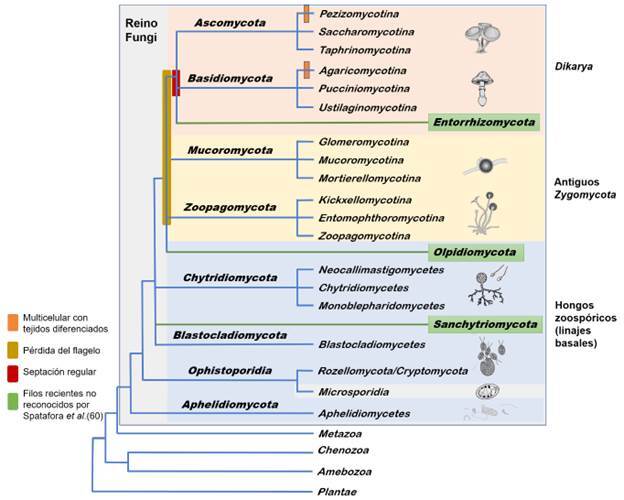
Árbol de la vida del reino Fungi

Por ser el más sólido, relativamente simple y ampliamente aceptado por los taxónomos, en esta revisión se presenta la descripción realizada en 2017, por Spatafora *et al.*^43^, del árbol de la vida del reino, haciendo algunas aclaraciones y adiciones que surgieron desde su publicación. Para complementar la información de los clados, también se utilizó la filogenia propuesta por Naranjo-Ortiz y Gabaldón en el 2019 ^24^. En la figura 1 se presenta una modificación de los árboles propuestos en estas filogenias.

Adaptado de Spatafora *et al.*^43^, Bauer *et al.*^59^, James *et al.*^62^, Li *et al.*^72^, Davis *et al.*^86^^)^ y Galindo *et al.*^114^. Las ramas del árbol representan la historia evolutiva de las especies, pero su longitud en esta gráfica no coincide con su cantidad de cambios. Se señalan los eventos más representativos en la evolución de los filos, como la pérdida del flagelo, septos regulares de las hifas y la formación de estructuras multicelulares con tejidos diferenciados. En los recuadros verdes, se presentan los tres filos no incluidos en la clasificación de Spatafora *et al.*^43^, pero aceptados por un gran número de taxónomos de hongos.

## 1. Hongos zoospóricos

Los hongos zoospóricos son el clado más primitivo del reino Fungi. Su morfología es simple al igual que su ciclo de vida, pero se evidencian diferencias importantes según los linajes ^64^. Dentro de este clado se incluyen: Opisthosporidia (Aphelidiomycota, Rozellomycota/Cryptomycota, Microsporidia), Blastocladiomycota y Chytridiomycota. Las relaciones evolutivas entre estos linajes siguen sin resolverse, dada la profunda divergencia entre estos, el muestreo incompleto actual y la naturaleza parasitaria de muchos de ellos ^24^.

La morfología de los hongos zoospóricos varía según el grado de desarrollo de su talo, el número y la posición de las estructuras reproductivas y su posición en el sustrato ^43^.

1.1. Opisthosporidia. También conocido como clado ARM, consiste en un superfilo propuesto por Karpov et al. ^65^ en el 2014, para incluir a tres filos de hongos zoospóricos: Aphelidiomycota, Rozellomycota-Cryptomycota y Microsporidia. Este superfilo se considera un brazo profundo del linaje Holomycota y todas las especies conocidas de este clado son parásitos intracelulares o parasitoides de una amplia gama de eucariotas.

1.1.1. Microsporidia. Incluye 1.440 especies y comprende un grupo diverso de parásitos intracelulares obligados de 16 filos de metazoos y 4 de protozoos. Fuera de este rango de huéspedes están poco descritos y algunos estudios ambientales sugieren una gran diversidad del grupo basada en su rango de huéspedes y su endemicidad ^66^.

Como consecuencia de su estilo de vida, presentan genomas muy reducidos (~2,000 genes que codifican para proteínas), hasta el punto de tener algunas características propias de los genomas procariotas, como el solapamiento de genes ^67^. A diferencia de los demás Opisthosporidia, los Microsporidia carecen de estructuras móviles y de verdaderas mitocondrias (con excepción de *Mitosporidium daphniae)*^66^; en su lugar, poseen unos orgánulos llamados mitosomas, sin genoma, y cuya función principal parece ser el ensamblaje de grupos de hierro y azufre. Muchos miembros de este clado han perdido la capacidad de realizar la glucólisis y el ciclo del ácido tricarboxílico, y dependen de la captación de ATP directamente de la célula huésped por medio de una serie de genes adquiridos horizontalmente. Presentan una estructura de penetración muy especializada en forma de arpón, denominada tubo polar, que es un aparato de Golgi modificado ^68^.

Ya que por mucho tiempo Microsporidia se consideraban protistas, las reglas de su nomenclatura siguen las convenciones para estos organismos. No obstante, desde el 2012, este grupo hace parte del reino Fungi ^56^. Dentro del filo, se han descrito cuatro géneros asociados con infección en humanos: *Encephalitozoon, Enterocytozoon, Nosema* y *Pleistophora,* que afectan más frecuentemente a pacientes infectados por el virus de la inmunodeficiencia humana (VIH), en quienes generan cuadros de diarrea crónica intermitente, colangiopatía, sinusitis, queratoconjuntivitis, nefritis y hepatitis ^69^.

1.1.2. Rozellomycota-Cryptomycota. El término Cryptomycota se propuso para describir una serie de organismos acuáticos cosmopolitas relacionados con Rozella. Debido a que *Cryptomyces* es un género de Ascomycota y no puede utilizarse para tipificar Cryptomycota, se sugiere abandonar este nombre y adoptar el de Rozellomycota. Rozella es un género de parasitoides flagelados de hongos zoospóricos (Chytridiomycota y Blastocladiomycota), oomicetos y algunas algas verdes ^70^.

A diferencia de Microsporidia, Rozella presenta un genoma no reducido y mitocondrias verdaderas con un metabolismo mitocondrial reducido.

Rozellomycota incluye cinco géneros: *Rozella, Paramicrosporidium, Nucleophaga, Morellospora* y *Mitosporidium*^71^. Se han encontrado secuencias ambientales filogenéticamente relacionadas con Rozella en casi todos los ambientes acuáticos, lo que comprende una divergencia de secuencias muy elevada ^70^^,^^71^.

1.1.3. Aphelidiomycota (Aphelidea). Este clado incluye hongos zoospóricos parasitoides intracelulares de algas ^24^. Los datos del transcriptoma muestran que los Aphelidiomycota tienen una composición genómica muy similar a la de los hongos de vida libre ^71^. Rozellomycota y Aphelidiomycota son morfológicamente similares entre sí, pero la distancia genética entre ellos es muy grande. El filo incluye una sola clase, Aphelidea, y tres géneros: *Aphelidium, Amoeboaphelidium* y *Pseudaphelidium*^65^^,^^71^.

1.2. Blastocladiomycota. Los dos filos restantes de hongos zoospóricos son Blastocladiomycota y Chytridiomycota. El orden de ramificación de estos dos linajes no está resuelto y ambos han sido propuestos en grandes análisis filogenéticos multigénicos y a escala genómica como linajes hermanos de los hongos terrestres no flagelados ^72^^-^^74^. Los Blastocladiomycota presentan una amplia gama de morfologías de crecimiento, desde monocéntricas con un desarrollo limitado del talo, hasta policéntricas con la producción de hifas robustas y coenocíticas. La mayoría de las especies conocidas presenta una verdadera alternancia generacional con fases de vida libre haploide y diploide ^43^.

Este filo tiene características más similares a las de los hongos terrestres, como hifas bien desarrolladas, mitosis cerrada, paredes celulares con β-1-3-glucano y Spitzenkórper ^75^. Contiene una única clase y un orden, Blastocladiomycetes y Blastocladiales, respectivamente. Los géneros más estudiados dentro de los Blastocladiales incluyen *Allomyces, Blastocladiella, Coelomomyces* y *Physoderma,* que presentan ecologías sapróbicas y parasitarias asociadas con animales y plantas ^43^.

1.3. Chytridiomycota (Chytridiomyceta). El filo Chytridiomycota para algunos autores está incluido dentro del subreino Chitridiomyceta, ya que en sus análisis el subfilo Neocallimastigomycota y la clase Monoblepharidiomycetes alcanzan el estatus de filo como clados hermanos de Chytridiomycota ^73^^,^^76^^,^^77^. No obstante, las filogenias de Spatafora *et al.*^43^ y Naranjo-Ortiz y Gabaldón ^24^, incluyen a Chytridiomycota dentro de sus tres clases Chytridiomycetes, Monoblepharidomycetes y Neocallimastigomycetes, que conforman colectivamente un clado monofilético bien sustentado en análisis a escala genómica, aunque la relación entre sí aún es incierta ^78^.

Los Chytridiomycota pudieron ser los primeros hongos en ambientes terrestres, pero no está claro si ciertos microfósiles precámbricos representan realmente especies del filo. Los Chytridiomycota son hongos que habitan en el agua, a menudo parásitos de algas y oomicetos, o habitantes del suelo, algunos de los cuales son parásitos de plantas vasculares. Unos pocos hongos quítridos parasitan huevos de animales y protozoos, mientras que otros son saprobios de restos vegetales en descomposición. Chytridiomycetes es, por mucho, la clase más grande de hongos zoospóricos, con alrededor de 1.000 especies descritas ^79^^,^^80^. Actualmente, *Batrachochytrium dendrobatidis* es el Chitridiomycete más conocido por su asociación con la extinción de algunos anfibios ^81^.

Monoblepharidomycetes, con sólo 30 especies en seis géneros ^43^, comprende un grupo de hongos zoospóricos, saprobios que crecen en agua dulce sobre ramitas y frutos sumergidos ^82^. Los Neocallimastigomycetes eran considerados como protozoos flagelados, pero ahora están situados en un grupo distinto de los quítridos centrales por sus secuencias de ADN ^73^; son los degradadores más potentes de celulosa y los únicos anaerobios obligados del reino fúngico que residen en el rumen y el tubo digestivo de los mamíferos más grandes, y de algunos reptiles, marsupiales y herbívoros aviares ^83^. Todas las especies producen celulasas y xilanasas que ayudan a degradar las fibras dietéticas de las paredes celulares de las plantas. Muchas de estas enzimas son de origen bacteriano y representan una transferencia horizontal de genes desde bacterias que presumiblemente coexisten con Neocallimastigales, en el tubo digestivo de los herbívoros ^84^. Carecen de mitocondrias verdaderas y albergan hidrogenosomas derivados de mitocondrias. Las especies de esta clase presentan genomas grandes (101 Mb en *Orpinomyces)* con gran contenido de elementos repetitivos y un contenido muy escaso de guaninas y citosinas (tan poco como 17 % en *Orpinomyces),* y un repertorio muy amplio y rico de enzimas que degradan carbohidratos ^84^.

A diferencia de cualquiera de los otros hongos verdaderos zoospóricos, los miembros de este filo pueden tener múltiples flagelos en una sola espora. De manera similar a Chytridiomycota, las formas pluricelulares de los Neocallimastigomycetes parecen carecer de una verdadera organización de hifas. La clase consta de unas 36 especies distribuidas en 20 géneros ^83^.

## 2. Ziqomicetos (mohos con septos)

El abandono del filo Zygomycota se formalizó por Hibbett *et al.* en el 2007 ^80^, porque las filogenias a escala genómica no apoyaban su monofilia y rechazan la zigospora como sinapomorfía ^74^. Los resultados de los estudios filogenéticos moleculares de ADNr y multilocus, resolvieron estos taxones en dos grandes grupos denominados zygomycetes I, que incluyó a Mucoromycotina, Mortierellomycotina y Glomeromycota ^73^; y zygomycetes II, que incorporó a Entomophthoromycota, Kickxellomycotina y Zoopagomycotina ^72^^,^^73^^,^^85^. La mayoría de los zigomicetos se caracterizan por tener hifas coenocíticas y reproducción asexual por esporangios, pero existen linajes caracterizados por hifas tabicadas o compartimentadas y reproducción asexual por formación de conidios.

La aparición de los zigomicetos marcó la pérdida del flagelo fúngico y el surgimiento de los hongos terrestres filamentosos. En las taxonomías más recientes, lo conforman dos linajes principales: uno compuesto por parásitos de opistocetos (Zoopagomycota) y otro que incluye simbiontes de plantas y saprótrofos (Mucoromycota) ^43^^,^^72^.

2.1. Zoopagomycota. Este clado, hermano de Mucoromycota y Dikarya, es el primer grupo divergente de hongos no flagelados y comprende tres subfilos: Zoopagomycotina, Kickxellomycotina y Entomophthoromycotina ^74^^,^^80^. Las ecologías primarias de los miembros del filo incluyen hongos patógenos y comensales de animales, parásitos de otros hongos y amebas, y otros raramente asociados con plantas. Los tres linajes tienen la capacidad de formar micelios verdaderos. No obstante, su agrupación es solo por afinidad filogenética y su estilo de vida asociado a metazoos, pues morfológicamente no comparten características.

El filo Zoopagomycotina contiene un único orden, Zoopagales, un clado monofilético que incluye cinco familias y alrededor de 20 géneros ^80^^,^^86^, caracterizados por hifas delgadas, coenocíticas y que forman haustorios sobre sus huéspedes o dentro de ellos. Los análisis moleculares de miembros de este subfilo sugieren altas tasas evolutivas ^86^.

Kickxellomycotina unifica varios grupos fúngicos poco estudiados, unidos por la presencia de micelios con septos que presentan poros únicos con un tapón lenticular y esporangios característicos, con cuatro órdenes, más varios géneros de ubicación filogenética aún no resuelta ^87^. Entomophthoromycotina comprende tres clases principales: Basidiobolomycetes, Neozygitomycetes y Entomophthoromycetes, cada una con un solo orden. Todos los grupos de Entomophthoromycotina presentan 24-metilcolesterol como el principal esterol de membrana ^88^.

Basidiobolomycetes es el linaje más antiguo y comprende el género *Basidiobolus,* un comensal intestinal saprótrofo de anfibios y reptiles, y patógeno oportunista humano ^89^. Entomophthoromycetes es la clase más rica en especies y la mejor caracterizada del subfilo con varias familias de parásitos de insectos, en su mayoría especializados, que forman un grupo monofilético bien definido, y algunos géneros pequeños compuestos por parásitos de algas desmídeas, nematodos, tardígrados y helechos. Comprende principalmente el género *Conidiobolus,* entidad saprobia, parásito facultativo de insectos y patógeno humano ocasional ^88^^,^^90^.

2.2. Mucoromycota. Es el grupo hermano de Dikarya, con estilos de vida dominantes asociados a plantas, lo que sugiere que el *Most Recent Common Ancestor* (MRCA) de Mucoromycota y Dikarya corresponde al origen de las asociaciones modernas hongo-planta. Comprende el grupo más grande y mejor estudiado de hongos zigomicetos y está formado por los subfilos: Glomeromycotina, Mortierellomycotina y Mucoromycotina ^74^^,^^91^.

A diferencia de Zoopagomycota, Mucoromycota se caracteriza por asociaciones vegetales y ecologías basadas en plantas (por ejemplo, micorrizas, endófitos radiculares, descomponedores, etc.). Algunos existen como parásitos de animales y otros hongos, pero todos ellos causan infecciones oportunistas en huéspedes con sistemas inmunológicos comprometidos o representan derivaciones relativamente recientes de ecologías sapróbicas ^92^.

Evidencia previa ubicaba al subfilo Glomeromycotina en un clado con Dikarya ^82^, por lo que llegó a considerarse un filo aparte ^93^. No obstante, las filogenias a escala genómica y los análisis del contenido de ADN apoyan firmemente a las micorrizas arbusculares como miembros de Mucoromycota y los relacionan con Mucoromycotina y Mortierellomycotina ^74^.

Los Mortierellomycotina son endófitos de las raíces de las plantas, pero se desconoce su efecto sobre la salud del huésped. El subfilo incluye una familia, 13 géneros y más de 100 especies reconocidas actualmente ^94^. Mortierellomycotina se diferencia de Mucoromycotina por la morfología de la zigospora y la ausencia de columela. Mucoromycotina contiene el resto de las especies conocidas de zigomicetos y se clasifica en tres órdenes: Mucorales, Umbelopsidales y Endogonales, y 17 familias ^76^^,^^92^^,^^95^.

Mucorales es uno de los grupos de hongos más comúnmente aislados porque muchos son colonizadores tempranos, de rápido crecimiento, de sustratos ricos en carbono. En comparación con la mayoría de los hongos, las paredes celulares de Mucoromycotina contienen quitosano, una forma desacetilada de la quitina, como principal componente estructural ^92^. También, presentan una extrusión del esporangióforo, denominada columela, que es sinapomórfica para el subfilo. La mayoría de las especies son saprótrofas y, ocasionalmente, pueden ser parásitos facultativos de animales, plantas y otros hongos. Incluyen especies de importancia clínica en humanos, donde son agentes patógenos oportunistas causantes de infecciones de rápida progresión y gran mortalidad ^96^.

2.3. Dikarya. Es el único subdominio descrito de los hongos y el grupo más rico en especies y mejor estudiado. Comprende los filos Ascomycota y Basidiomycota, aunque recientemente se ha propuesto un tercer grupo de endófitos radiculares: Entorrhizomycota ^59^.

El nombre Dikarya se refiere a la condición de poseer dos núcleos genotípicamente distintos dentro del talo en algún momento del ciclo de vida. Las hifas de Dikarya están regularmente tabicadas, el ergosterol es el principal esterol de la membrana y varios linajes son capaces de formar estructuras reproductivas o vegetativas multicelulares. Se han secuenciado más genomas de especies de Ascomycota y Basidiomycota que de otros filos del reino Fungi (http://genome.jgi.doe.gov/fungi/), lo que ha contribuido a una mayor resolución de las relaciones filogenéticas y a los procesos evolutivos que han dado forma a la diversidad filogenética y ecológica de Dikarya.

2.4. Ascomycota. Es el filo fúngico más grande y comprende, aproximadamente, dos tercios de todas las especies descritas ^97^. Incluye descomponedores de múltiples sustratos (por ejemplo, estiércol, madera, suelo), simbiontes y asociados de plantas y animales, habitantes de ecosistemas marinos y terrestres, y la mayoría de las especies patógenas para humanos ^98^. Ascomycota consta de tres subfilos: Taphrinomycotina, y los linajes hermanos Saccharomycotina y Pezizomycotina.

Durante la reproducción sexual, se induce la formación de hifas dicariontes de vida muy corta, que conducen a la formación del asca, una estructura en forma de saco que contiene las ascosporas (normalmente ocho) derivadas de la meiosis. Las esporas asexuales (conidias) u otros medios de propagación asexual son muy comunes, y los estadios sexuales son desconocidos para muchos miembros del filo. El filo incluye desde simples levaduras a hongos con cuerpos fructíferos macroscópicos muy complejos ^43^. Taphrinomycotina (antes conocida como Archiascomycota) forma el primer clado divergente de Ascomycota reconocido por la gran diversidad morfológica entre sus géneros, con formas de crecimiento tanto levaduriformes como filamentosas, y la monotípica de sus clases (un solo género por clase) ^99^.

El subfilo Saccharomycotina (anteriormente conocido como Hemiascomycota) incluye una sola clase y un orden (Saccharomycetes, Saccharomycetales), que abarca 14 familias y alrededor de 1.500 especies ^100^. Es el linaje eucariota mejor representado en términos de información genómica con *Saccharomyces cerevisiae* como el primer eucariota secuenciado y 156 especies con secuencia genómica completa (cuadro 1).

Los Saccharomycetales incluyen la mayoría de las levaduras de ascomicetos y se caracterizan por la gemación en la reproducción asexual ^100^. Aunque son relativamente similares en morfología, los datos genómicos de las últimas décadas han permitido abordar la problemática filogenia y taxonomía de las levaduras ^101^. Se ha demostrado la parafilia de ciertos géneros importantes -por ejemplo, *Candida-* reflejado en el desplazamiento de varias especies de *Candida,* de importancia clínica, a otros como *Pichia, Meyerozyma* y *Nakaseomyces,* entre otros ^102^.

Los miembros del subfilo Pezizomycotina (anteriormente conocido como Euascomycota) son en su mayoría filamentosos y anastomosados, con septos que presentan el cuerpo de Woronin, aunque muchos tienen crecimiento dimorfo, y otros son unicelulares. En comparación con otros hongos, tienden a contener abundantes enzimas para el metabolismo secundario ^103^. El subfilo contiene aproximadamente 63.000 especies que se clasifican en 13 clases y 67 órdenes ^43^^,^^80^. No obstante, una fracción importante permanece sin clasificar, tal y como se describe en el catálogo de la vida ^104^. Contiene más de 5.000 especies, cuya afiliación a cualquiera de estas clases es desconocida y muchas ni siquiera están clasificadas a nivel de familia.

2.5. Basidiomycota. Es el segundo filo más rico en especies de hongos, con cerca de 32.000 descritas ^80^^,^^105^, las cuales presentan una amplia gama de estilos de vida y estrategias de organización celular.

Este filo incluye los hongos más complejos en términos de ciclo celular y multicelularidad, y su principal característica es la producción de células especializadas en forma de clava, llamadas basidias, que suelen producir cuatro esporas sexuales, característica que es compartida con Entorrhizomycota ^106^. El apareamiento suele implicar la anastomosis entre las hifas de cada hongo (somatogamia) y la formación de un dicarión, condición mantenida gracias a la formación de fíbulas. Los núcleos del dicarión permanecen asociados antes de sufrir la cariogamia y la meiosis para producir las basidiosporas. Las hifas, cuando están presentes, son tabicadas y en algunos grupos los septos presentan doliporos (un retículo endoplasmático modificado en forma de paréntesis).

El filo contiene cuatro linajes bien definidos: Pucciniomycotina, Ustilagomycotina, Agaricomycotina, y el recientemente descrito, Wallemiomycotina ^*107*^. Pucciniomycotina incluye más de 8.400 especies descritas, clasificadas en 10 clases, 20 órdenes y 35 familias, en las que son comunes las levaduras y las formas de crecimiento dimorfo ^108^. Este subfilo contiene un clado muy diverso de agentes patógenos vegetales llamados royas (clase Pucciniomycetes), así como algunas especies de vida libre que suelen crecer como levaduras saprótrofas. También, se describen micoparásitos, patógenos de insectos y micorrizas ^106^. Algunos miembros del grupo, en particular en Pucciniomycetes, tienen genomas muy grandes ^109^, y varios tienen ciclos de vida muy complejos que implican varios huéspedes y etapas de vida libre. La pared celular contiene manosa, pero carece de xilosa.

El subfilo Ustilaginomycotina comprende unas 1.700 especies de levaduras, casi todas anamorfas o dimorfas. La mayoría de las especies descritas son agentes patógenos vegetales, normalmente biotróficos (tizones), mientras que otras viven como levaduras saprótrofas de vida libre o patógenos animales (por ejemplo, *Malassezia).* Los hongos patógenos de plantas suelen tener estados asexuales de levadura, a menudo con capacidad sapróbica, y un estado micelial dicarionte infectante ^106^.

Agaricomycotina es el grupo más grande de Basidiomycota, que contiene alrededor de dos tercios de todos los *Basidiomycota* descritos. El subfilo tiene tres clases: Tremellomycetes, Dacrymycetes y Agaricomycetes ^80^.

Los Tremellomycetes incluyen especies de levaduras como *Cryptococcus* y *Tremella*^110^. Muchas especies son micoparásitas, viven dentro de los cuerpos fructíferos de otros hongos y los infectan por medio de un tipo particular de haustorio; mientras otras son levaduras de vida libre o patógenos animales ^111^. Dacrymycetes incluye un pequeño grupo de hongos descomponedores de la madera ^112^, mientras que Agaricomycetes son los hongos predominantes en los bosques con 22 órdenes y más de 21.000 especies ^106^. Son principalmente simbiontes ectomicorrícicos, hongos patógenos de los árboles y agentes de la descomposición de la madera y la hojarasca, que provocan podredumbres pardas o blancas.

## **3. Filos recientes no incluidos por Spatafora *et al.*** ^43^

Es importante anotar que constantemente se proponen nuevos grupos taxonómicos, inclusive en niveles superiores de filo o subfilo, dada la evidencia derivada de los análisis filogenéticos, algunos con datos que dan peso a los hallazgos. No obstante, muchos de estos no tienen eco en los taxónomos de hongos y terminan en el olvido o a la espera de nuevos estudios que refuercen los datos originales. Tres nuevos filos, recientemente propuestos, aparecen con frecuencia en los árboles filogenéticos: Entorrhizomycota, Sanchytriomycota y Olpidiomycota.

3.1. Entorrhizomycota. Una de las clasificaciones más controvertidas en el árbol de la vida de los hongos es la elevación de Entorrhiza, un parásito de la raíz formador de agallas en Poales, al nivel de filo. Este taxón ha pertenecido clásicamente, junto con Ustilaginomycotina, a Basidiomycota por sus grandes similitudes en la ultraestructura del poro del septo y la germinación de teliosporas.

El filo Entorrhizomycota se erigió después de que una filogenia de cinco genes colocara a Entorrhiza como hermana de los otros Dikarya, aunque otros análisis posteriores lo propusieran dentro o como hermana de Basidiomycota ^59^. Debido a las similitudes morfológicas con los tizones, el bajo número de genes empleados en la filogenia y el escaso apoyo estadístico, esta ubicación es controvertida. En el 2017, un análisis de seis genes publicado por Zhao *et al.*^105^, y que recibió gran apoyo, erigió el nuevo filo Entorrizhamycota, luego consolidado por análisis posteriores ^113^. El filo incluye dos órdenes, Entorrhizales (con 16 especies) y Talbotiomycetales (con una sola especie), pero estudios ambientales sugieren la carencia de diversidad en este filo ^113^.

3.2. Nuevos filos zoospóricos. El avance en las técnicas de secuenciación a partir de una sola célula está permitiendo estudiar hongos poco conocidos, principalmente de los clados basales. Con los recientes hallazgos obtenidos por estas metodologías, se ha propuesto la existencia de dos nuevos filos dentro de los hongos zoospóricos: Sanchytriomycota y Olpidiomycota ^114^.

3.2.1. Sanchytriomycota. El análisis filogenético de dos conjuntos de datos independientes de proteínas conservadas demostró que Sanchytriomycota forma un nuevo filo fúngico de rápida evolución, hermano de Blastocladiomycota. Este filo solo comprende dos especies de hongos atípicos, parásitos de algas *(Amoeboradix gromovi* y *Sanchytrium tribonematis),* con un ciclo vital complejo que incluye una fase flagelada (zoosporas) con flagelos inmóviles que participan en un proceso reductor continuo ^114^.

3.2.2. Olpidiomycota. Los análisis filogenéticos confirmaron la posición del flagelado *Olpidium* como hermano del principal grupo de hongos no flagelados (Zoopagomycota, Mucoromycota y Dikarya) ^115^, lo que sugiere que, de forma análoga a los sanquítridos, los Olpidiomycota representan un estadio intermedio entre los hongos zoospóricos que poseen flagelos completamente funcionales y los hongos que carecen de ellos. El filo incluye una sola clase, un solo orden (Olpidiales) y una sola familia, cuatro géneros y alrededor de 30 especies ^116^. El género más reconocido es *Olpidium,* compuesto por hongos zoospóricos, endoparásitos morfológicamente simples con una pared celular (presumiblemente quitinosa) presente en todas las fases del crecimiento parasitario. Se encuentra como endoparásito obligado de algas, plantas, hongos y pequeños animales ^114^.

## Conclusiones

La organización taxonómica de los hongos continúa siendo una asignatura pendiente. Son muchos los esfuerzos llevados a cabo desde siglos atrás para conseguir un árbol de la vida del reino Fungi coherente, pero, a pesar de la plétora de datos morfológicos, bioquímicos, fisiológicos, ecológicos, y más recientemente, moleculares, el camino por recorrer es largo, y con toda seguridad, lleno de tropiezos, retrocesos, y por qué no, algunos atajos gracias a los nuevos desarrollos que puedan producirse en los próximos años en cuanto a la obtención, manipulación y organización de la información.

Sin duda, si los investigadores de la taxonomía y la filogenia de los hongos no llegan a un acuerdo sobre cómo trabajar mancomunadamente, la tarea será más difícil. Es necesaria la creación de unas pocas bases de datos curadas para los genomas y secuencias incluidas en los diferentes estudios filogenéticos (un número extenso continuaría con la disgregación y la confusión). Además, deben depurarse las bases de datos existentes para eliminar o completar aquellas secuencias que generan conflicto cuando son incluidas en los análisis filogenéticos. El acceso a la información en el área debería ser libre para que los científicos de países con escasos recursos, con zonas muy endémicas de hongos no descubiertos y que son piezas clave en el rompecabezas del árbol de la vida fúngico, puedan generar datos filogenéticos de gran calidad.

Uno de los mayores problemas para los que desean aportar en el estudio de la taxonomía fúngica, es encontrarse con los desacuerdos entre los grupos más reconocidos que trabajan en el área. Estas disputas solo generan desasosiego en los investigadores y perpetúan las publicaciones redundantes o que poco aportan a la organización taxonómica de los organismos vivos. Las metodologías basadas en las ciencias ómicas facilitan la identificación de nuevos taxones y permiten trabajar con un número muy grande de secuencias. Estas herramientas, sumadas a las nuevas técnicas o los métodos de análisis que se propongan en los años venideros, ayudarán para la creación de reglas claras sobre cómo abordar la taxonomía fúngica, definir los alcances y los límites de los análisis, entender cómo delimitar una especie dentro del reino Fungi y decidir hacia dónde se deben dirigir los mayores esfuerzos.

Para los profesionales del área de la salud, la taxonomía de los hongos puede resultar tediosa o poco relevante para su quehacer diario. No obstante, estos análisis son el insumo para identificar a los hongos, entender su biología, comprender su papel en los diferentes ecosistemas y reconocer su impacto cuando asumen nuevos roles como contaminantes o causantes de procesos infecciosos en humanos, animales y plantas. Son precisamente los estudios metagenómicos, junto con los análisis filogenéticos, los que han permitido reconocer a los hongos del microbioma humano y entender que, más que simples comensales, participan de manera activa en la homeostasis de los tejidos donde se encuentran. También, los análisis del genoma ayudan a indagar sobre la epidemiología de las micosis, los procesos alérgicos y sus agentes causales, y a definir si un brote está asociado a un ambiente hospitalario o extrahospitalario, según la posible fuente de contaminación.

La secuenciación de los genomas de un mayor número de taxones dará las herramientas para:

1. diseñar nuevas técnicas de diagnóstico molecular, con mayor sensibilidad, especificidad y rápida ejecución;
2. facilitar el desarrollo de nuevos fármacos antifúngicos y métodos terapéuticos alternativos, y
3. reconocer los mecanismos que expliquen la disminución o no de la respuesta a la terapia antifúngica asociada a mecanismos de resistencia innatos o adquiridos.
